# Mitochondrial Biogenesis in Response to Chromium (VI) Toxicity in Human Liver Cells

**DOI:** 10.3390/ijms18091877

**Published:** 2017-09-14

**Authors:** Xiali Zhong, Rita de Cássia da Silveira e Sá, Caigao Zhong

**Affiliations:** 1 Department of Health Toxicology, School of Public Health, Central South University, Changsha 410008, China; xzhong16@jhu.edu; 2 Department of Physiology and Pathology, Health Sciences Center, Federal University of Paraíba, João Pessoa 58059-900, Brazil; ritacassia.sa@bol.com.br

**Keywords:** mitochondrial biogenesis, hexavalent chromium, mitochondrial DNA, HepG 2 cells

## Abstract

Hexavalent chromium (Cr(VI)) is a ubiquitous environmental pollutant, which poses a threat to human public health. Recent studies have shown that mitochondrial biogenesis can be activated by inflammatory and oxidative stress. However, whether mitochondrial biogenesis is involved in Cr(VI)-induced hepatotoxicity is unclear. Here, we demonstrated the induction of inflammatory response and oxidative stress, as indicated by upregulation of inflammatory factors and reactive oxygen species (ROS). Subsequently, we demonstrated that mitochondrial biogenesis, comprising the mitochondrial DNA copy number and mitochondrial mass, was significantly increased in HepG2 cells exposed to low concentrations of Cr(VI). Expression of genes related to mitochondrial function complex I and complex V was upregulated at low concentrations of Cr(VI). mRNA levels of antioxidant enzymes, including superoxide dismutase 1 and 2 (*SOD1* and *SOD2*, respectively), kech like ECH associate protein 1 (*KEAP1*) and nuclear respiratory factor 2 (*NRF-2*), were also upregulated. Consistent with the above results, mRNA and protein levels of key transcriptional regulators of mitochondrial biogenesis such as the peroxisome-proliferator-activated receptor γ coactivator-1α (PGC-1α), NRF-1 and mitochondrial transcription factor A (TFAM) were increased by low concentrations of Cr(VI) in HepG2 cells. Moreover, we found that PGC-1α and NRF-1 tended to translocate into the nucleus. The expression of genes potentially involved in mitochondrial biogenesis pathways, including mRNA level of silent information regulator-1 (*SIRT1*), forkhead box class-O (*FOXO1*), threonine kinase 1 (*AKT1*), and cAMP response element-binding protein (*CREB1*), was also upregulated. In contrast, mitochondrial biogenesis was inhibited and the expression of its regulatory factors and antioxidants was downregulated at high and cytotoxic concentrations of Cr(VI) in HepG2 cells. It is believed that pretreatment with α-tocopherol could be acting against the mitochondrial biogenesis imbalance induced by Cr(VI). In conclusion, our study suggests that the homeostasis of mitochondrial biogenesis may be an important cellular compensatory mechanism against Cr(VI)-induced toxicity and a promising detoxification target.

## 1. Introduction

Hexavalent chromium (Cr(VI)) is widely distributed in the environment and commonly used in industries. It has been demonstrated that Cr(VI) can cause lung cancer through occupational exposure to contaminated air [1]. However, the general population is exposed to Cr(VI) through consumption of contaminated drinking water, which results in severe public health issues [2]. The United State National Toxicology Program conducted short-term and long-term toxicity and carcinogenicity studies on Cr(VI) in drinking water, and reported that Cr(VI) caused oral and small intestine cancer, and liver injury in rodents [3]. Human oral exposure to Cr(VI) has been shown to produce hepatotoxicity and provided evidence that Cr(VI) accumulation occurs mostly in the liver—The largest detoxification organ by oral exposure route [4,5]. Moreover, Cr(VI) may cause primary liver cancer and increase the risk of deterioration of cancer patients [6,7]. The mechanism of Cr(VI)-induced hepatotoxicity is still not fully understood. Previous studies indicated that Cr(VI) could cause liver damage through the formation of DNA double-strand breaks and Cr-DNA adducts in the nuclear DNA [4]. It has also been shown that mitochondrial dysfunction could be caused by Cr(VI) exposure: Cr(VI) could reduce mitochondrial DNA (mtDNA) copy number and inhibit mitochondria electron transport chain complex I, resulting in perturbation of mitochondrial respiration and redox homeostasis [8,9]. These reports indicate that mitochondrion is one of the most sensitive targets of Cr(VI) toxicity.

Mitochondrial biogenesis is among the self-repairing and compensatory mechanisms that can be activated by cellular stress and inflammatory response or in response to environmental stimuli to maintain energetic and redox homeostasis [10]. Cell respiratory defects and reactive oxygen species (ROS) production can activate mitochondrial biogenesis as an adaptive strategy to respond to diverse mitochondrial disturbances [11]. One main regulator of mitochondrial biogenesis is the peroxisome-proliferator-activated receptor γ coactivator-1α (PGC-1α) [12], which is modulated by the binding of cAMP response element-binding protein (CREB), the activating transcription factor (ATF2), and the forkhead box class-O (FOXO1) [13]. PGC-1α interacts with CREB and several other transcriptional factors to control other downstream factors, including nuclear respiratory factor 1 (NRF-1) and NRF-2, and to regulate the subsequent transcription of multiple genes involved in mitochondrial biogenesis and energy metabolism [14]. Mitochondrial biogenesis takes place as part of the normal cell proliferation process to maintain the increasing cell mass in general conditions. When it encounters cellular stress, mitochondrial biogenesis is extended to sustain ATP supply and to keep up with the increasing energy demand [15]. PGC-1α, NRF-1 and NRF-2 are primarily localized in the cytoplasm under general physiological conditions. However, in response to stress, nuclear genes related to mitochondrial biogenesis translocate to the nucleus and bind to downstream transcription factors to process their transcriptional activity. Consequently, transcription of genes related to mitochondrial biogenesis is initiated [16,17], and includes an increase of mitochondrial transcription factor A (TFAM) [18], which functions to replicate and transcribe mtDNA, promoting mitochondrial biogenesis, and possibly packing and repairing of mtDNA to prevent damage [19].

It has been well established that Cr(VI) can disrupt mitochondrial function in liver cells. However, whether mitochondrial biogenesis is involved in Cr(VI)-induced hepatotoxicity is unclear. In the present study, we used Human Hepatocellular Carcinoma cells (HepG2) as a model to investigate the effect of Cr(VI) on mitochondrial biogenesis.

## 2. Results

### 2.1. Cr(VI) Induces Cytotoxicity and Cell Death in HepG2 Cells

**Figure 1:**
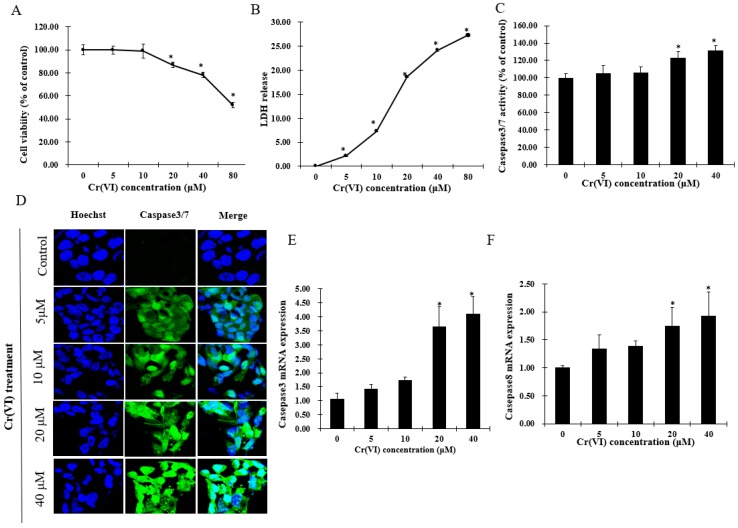
Hexavalent chromium (Cr(VI)) induces cytotoxicity and cell death of HepG2 cells. (**A**) Cell viability of HepG2 cells exposed to Cr(VI) for 24 h was performed by resazurin reduction assays; (**B**) lactate dehydrogenase (LDH) release of HepG2 cells exposed to Cr(VI) for 24 h was evaluated with LDH assays; (**C**) percentage of caspase-3/7 positively stained cells were normalized with the total nuclei count by using Image-J software; (**D**) caspase-3/7 activity of HepG2 cells exposed to Cr(VI) for 24 h. Green signals located in the nuclei indicate caspase-3/7 activation. Nuclei were stained with Hoechst 33342. Images were captured with confocal microscope (Zeiss, Germany), scale bar 10 μm; (**E**,**F**) mRNA expression level of caspase-3 and caspase-8 was detected by real-time qPCR. Data are presented as mean ± SME of the values obtained in three independent experiments, each using triplicate cultures. After outlier analysis, the number of values used in the calculation of the corresponding mean varied from six to nine. * Compared with control; * *p* < 0.05.

Cytotoxicity experiments were performed to estimate sub-cytotoxic concentrations of Cr(VI) by determining cell viability and lactate dehydrogenase (LDH) release. After exposure to Cr(VI) for 24 h, HepG2 cells did not exhibit any change in cell viability at concentrations lower than 10 μM. However, the cell viability was significantly decreased in a concentration-dependent manner over a range of Cr(VI) concentrations between 20 and 80 μM. In the LDH release assay, Cr(VI) significantly and concentration-dependently increased LDH release. Apoptotic cell death was confirmed by increased activation of caspases-3/7 and caspase-8 in HepG2 cells exposed to Cr(VI) at different concentrations. Apoptotic cells with activated caspase-3/7 exhibited bright green nuclei, whose intensity increased with increasing concentrations of Cr(VI) (Figure 1D). The mRNA levels of caspase-3 and caspase-8 were consistent with the caspase-3/7 staining change. Based on the results above, it is believed that Cr(VI) induced cytotoxicity and cell death in HepG2 cells, and that these cells could tolerate Cr(VI)-induced insult at lower range concentrations up to 10 μM.

### 2.2. The Effect of Cr(VI) on the mRNA Level of Inflammatory Cytokines and Redox Reaction in HepG2 Cells

**Figure 2:**
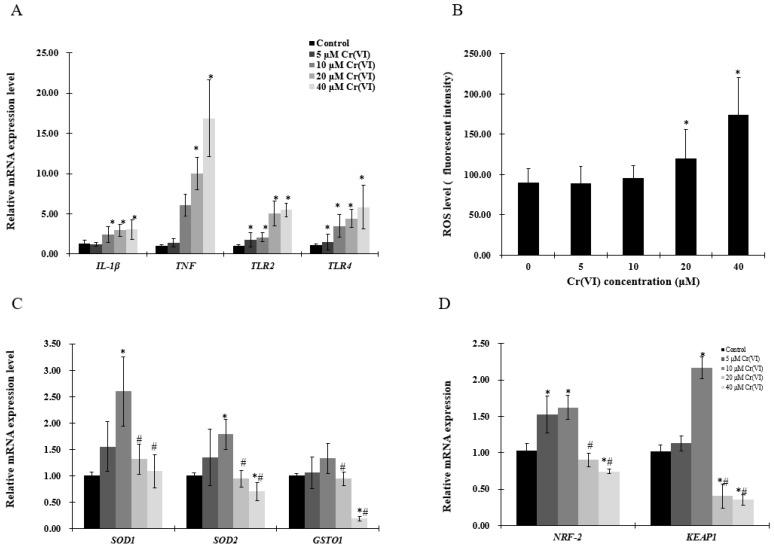
Effects of Cr(VI) on inflammatory cytokines and antioxidant enzymes in HepG2 cells. (**A**,**C**,**D**) mRNA expression levels of genes were detected by real-time qPCR; and (**B**) reactive oxygen species (ROS) intensity was detected by using CellROX**^®^** Green reagent. Data are presented as means ± SME of the values obtained in three independent experiments, each using triplicate cultures. After outlier analysis, the number of values used in the calculation of the corresponding mean varied from six to nine. * Compared with control, * *p* < 0.05; # compared with 2 μM of Cr(VI) treatment group, # *p* < 0.05. Abbreviations: glutathione S-transferase Ω 1, *GSOT1*; interleukin 1-β, IL-1β; kelch like ECH associate protein 1, *KEAP1*; nuclear respiratory factor 2, *NRF-2*; reactive oxygen species, ROS; superoxide dismutase, *SOD*; tumor necrosis factor, TNF; toll-like receptor 2/4, *TLR2/4*.

The mRNA levels of tumor necrosis factor (*TNF*) and interleukin 1-β (*IL-1β* ) were increased in a concentration-dependent manner (Figure 2A,B). As pro-inflammatory cytokines are driven by toll-like receptor (TLR)-mediated signals, *TLR2* and *TLR4* expression was examined by real-time qPCR, which showed that both *TLR2* and *TLR4* mRNA levels were significantly upregulated (Figure 2A). Cells stained with CellROX**^®^** Green and analyzed by a plate reader revealed that Cr(VI) increased ROS levels in a concentration-dependent manner (Figure 2B). However, the antioxidant enzymes superoxide dismutase (*SOD1*, *SOD2*), glutathione S-transferase O 1 (*GSOT1*), nuclear respiratory factor 2 (*NRF-2*) and kelch like ECH associate protein 1 (*KEAP1*) mRNA levels were upregulated at Cr(VI) concentrations up to 10 μM, which tended to decrease at 20 μM (Figure 2C,D).

### 2.3. Exposure to Low Concentrations of Cr(VI) Induces Adaptive Upregulation of Mitochondrial Biogenesis

**Figure 3:**
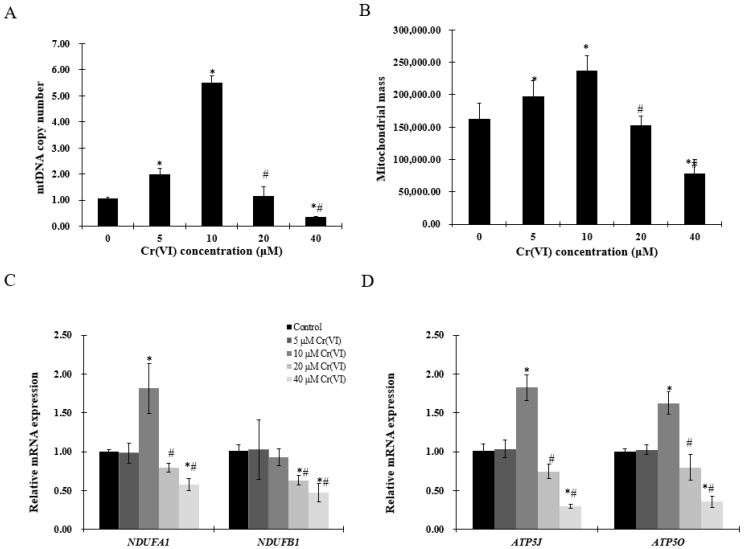
Effects of Cr(VI) on mitochondrial biogenesis and mitochondrial function. (**A**) mtDNA copy number was measured by real-time qPCR; (**B**) mitochondrial mass of HepG2 cells exposed to Cr(VI) was detected by 10-*N*-nonyl acridine orange (NAO) fluorescence intensity; (**C**,**D**) mRNA expression levels of genes related to mitochondrial function were detected by real-time qPCR. Data are presented as means ± SME of the values obtained in three independent experiments, each using triplicate cultures. After outlier analysis, the number of values used in the calculation of the corresponding mean varied from six to nine. * Compared with control, * *p* < 0.05; # compared with 2 μM of Cr(VI) treatment group, # *p* < 0.05. Abbreviations: ATP synthase, H+ transporting, mitochondrial Fo complex subunit F6, *ATP5J*; ATP synthase, H+ transporting, mitochondrial F1 complex, O subunit, *ATP5O*; NADH: ubiquinone oxidoreductase subunit A1, *NDUFA1*; NADH: ubiquinone oxidoreductase subunit B1, *NDUFB1*; mitochondrial DNA, mtDNA.

To evaluate mitochondrial biogenesis, mtDNA copy number and mitochondrial mass were examined. Increase in mtDNA copy number and mitochondrial mass was observed in HepG2 cells exposed to up to 10 μM of Cr(VI), followed by a decrease at 20 μM (Figure 3A,B). Mitochondrial function was assessed by complex I (NADH: ubiquinone oxidoreductase subunit A1 (NDUFA1) or NADH: ubiquinone oxidoreductase subunit B1 (NDUFB1)) and complex V (ATP synthase, H+ transporting, mitochondrial Fo complex subunit F6 (ATP5J) or ATP synthase, H+ transporting, mitochondrial F1 complex, O subunit (ATP5O)). The mRNA level of *NDUFA*1, but not of *NDUFB1*, was significantly increased at concentrations up to 10 μM of Cr(VI), while the highest concentration produced statistically significant decrease of both *NDUFA1* and *NDUFB1*. *ATP5J* and *ATP5O* mRNA expression was upregulated at concentrations up to 10 μM of Cr(VI), but decreased at 20 μM (Figure 3D).

### 2.4. Upregulation of PGC-1α Expression and Its Downstream Targets at Low Concentrations of Cr(VI)

**Figure 4:**
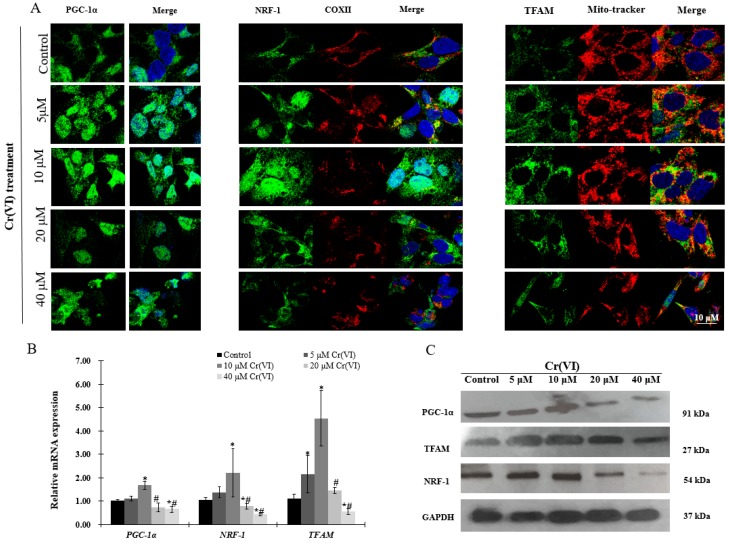
Effects of Cr(VI) on mitochondrial biogenesis regulatory factors in HepG2 cells. (**A**) PGC-1α (green) expression and location of HepG2 cells exposed to Cr(VI) for 24 h were observed by immunocytochemistry staining; co-immunostaining of NRF-1 (green) with COX II (red); co-immunostaining of TFAM (green) with Mito-tracker (red). Nuclei were stained with Hoechst 333432. Images were obtained by confocal microscopy (63×), scale bar: 10 μm; (**B**) mRNA expression levels of PGC-1α, *NRF-1* and *TFAM* were detected by real-time qPCR; (**C**) protein expression levels of PGC-1α, NRF-1 and TFAM were examined by Western blot analysis. Data are presented as mean ± SME of the values obtained in three independent experiments, each using triplicate cultures. After outlier analysis, the number of values used in the calculation of the corresponding mean varied from six to nine. * Compared with control, * *p* < 0.05; # compared with 2 μM of Cr(VI) treatment group, # *p* < 0.05. Abbreviations: cytochrome c oxidase subunit 2, COX II; nuclear respiratory factor 1, NRF-1; peroxisome-proliferator-activated receptor γ coativator-1 α, PGC-1α; mitochondrial transcription factor A, TFAM; glyceraldehyde 3-phosphate dehydrogenase, GAPDH.

Immunofluorescence, real-time qPCR and Western blot analyses were performed to investigate the expression of PGC-1α and its downstream targets, which regulate mitochondrial biogenesis. There was a concentration-dependent increase in PGC-1α translocation into the nucleus after exposure to up to 10 μM of Cr(VI) for 24 h. Afterwards, the response declined and became fragmented at the highest Cr(VI) concentration (Figure 4A). NRF-1 exhibited similar translocation from the cytoplasm into the nucleus, while the expression of COX II showed no changes after exposure to Cr(VI) (Figure 4A). The fluorescence of TFAM was increased and localized in the mitochondria after exposure to 5 or 10 μM of Cr(VI) for 24 h, but declined at 20 μM. Interestingly, exposure to 40 μM of Cr(VI) caused mitochondrial filament elongation, and both mitochondria and TFAM were fragmented and localized to the nuclear perimeter (Figure 4A). The mRNA and protein expression levels of PGC-1α, NRF-1, and TFAM were upregulated, reaching a peak at 10 μM of Cr(VI), which declined thereafter (Figure 4B,C).

### 2.5. Expression of Genes Involved in Mitochondrial Biogenesis Pathway after Exposure to Cr(VI) in HepG2 Cells

**Figure 5:**
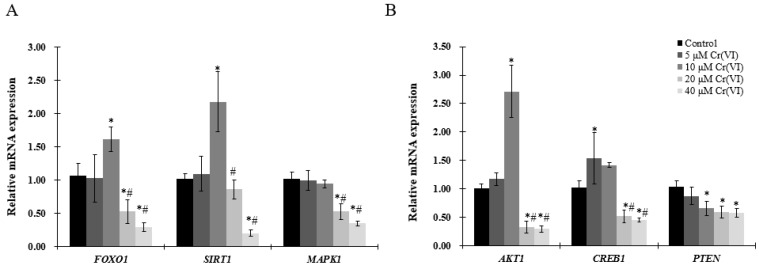
Effects of Cr(VI) on genes involved in regulatory pathways of mitochondrial biogenesis in HepG2 cells. mRNA expression levels were detected by real-time qPCR. (**A**) gene expression of *FOXO1*, *SIRT1*, and *MAPK1*; (**B**) gene expression of *AKT1*, *CREB1*, and *PTEN*. Data are presented as mean ± SME of the values obtained in three independent experiments, each using triplicate cultures. After outlier analysis, the number of values used in the calculation of the corresponding mean varied from six to nine. * Compared with control, * *p* < 0.05; # compared with 2 μM of Cr(VI) treatment group, # *p* < 0.05. Abbreviations: threonine kinase 1, *AKT1*; forkhead box class-O, *FOXO1*; mitogen-activated protein kinase 1, *MAPK1*; silent information regulator-1, *SIRT1*; phosphatase tensin homolog, *PTEN*.

To better understand the mechanism of mitochondrial biogenesis activation by low concentration of Cr(VI), genes involved in mitochondrial biogenesis pathways were analyzed by real-time qPCR. mRNA expression of *FOXO1*, *SRIT1*, *AKT1* and *CREB1* was similarly increased, reaching a peak at 10 μM of Cr(VI), but showing a tendency to decrease thereafter (Figure 5A,B). *MAPK1* and *PTEN* mRNA levels were decreased in a concentration-dependent manner (Figure 5A,B), without exhibiting any significant change at low concentrations of Cr(VI).

### 2.6. The Effect of α-Tocopherol on Mitochondrial Biogenesis in HepG2 Cells Exposed to Cr(VI)

**Figure 6:**
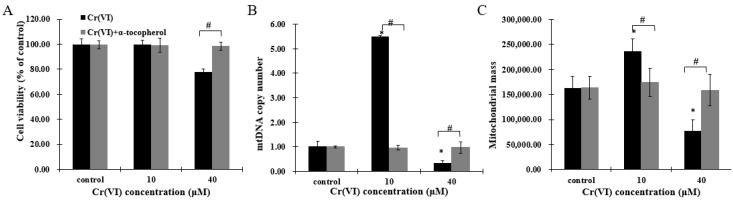

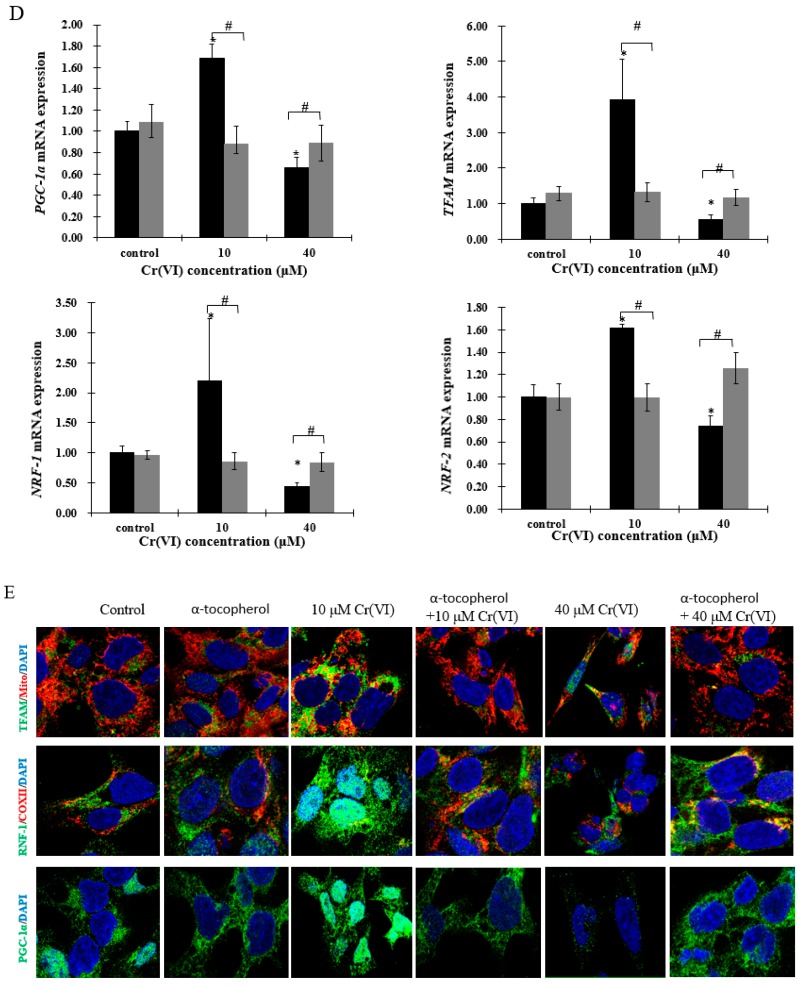
Cr(VI) perturbed mitochondrial biogenesis, an effect restored by α-tocopherol in HepG2 cells. (**A**) Cell viability was assessed by resazurin reduction assays; (**B**) mtDNA copy number was measured by real-time qPCR; (**C**) mitochondrial mass of HepG2 cells exposed to Cr(VI) was detected by NAO fluorescence intensity; (**D**) gene expression related to mitochondrial biogenesis was quantified by real-time qPCR; (**E**) the **upper** panels show images of cells co-incubated with TFAM (green) and Mito-tracker (red); the **middle** panels show images of cells co-incubated with NRF-1 (green) and COXII (red); and the **lower** panels show images of PGC-α (green). Images were captured by confocal microscopy with a 63× objective, scale bar: 10 μm. Data are presented as mean ± SME of the values obtained in three independent experiments, each using triplicate cultures. After outlier analysis, the number of values used in the calculation of the corresponding mean varied from six to nine. * Compared with control, * *p* < 0.05; # Compared with Cr(VI) treatment group, # *p* < 0.05. Abbreviations: nuclear respiratory factor 1, NRF-1; peroxisome-proliferator-activated receptor γ coativator-1 α, PGC-1α; mitochondrial transcription factor A, TFAM.

As Cr(VI) disturbed mitochondrial biogenesis and induced redox imbalance, HepG2 cells were pretreated with antioxidant α-tocopherol to investigate whether this component could protect mitochondrial biogenesis. After incubation with 50 μM of α-tocopherol 2 h before Cr(VI) exposure, cell viability was restored (Figure 6A). Furthermore, mitochondrial biogenesis, comprising recovering mtDNA copy number and mitochondrial mass, and the mitochondrial biogenesis regulators PGC-1α, NRF-1, NRF-2 and TFAM were maintained in a normal range (Figure 6B–E). These results indicate that α-tocopherol can regulate mitochondrial biogenesis to resist Cr(VI) toxicity, and that mitochondrial biogenesis may be a sensitive target of Cr(VI) poisoning and detoxification.

## 3. Discussion

Presence of Cr(VI) in drinking water is a major public health concern worldwide. Drinking water contaminated with Cr(VI) causes liver injury and may increase the risk of primary liver cancer. In this study, we found that LDH release started at 5 μM of Cr(VI) without changing cell viability until reaching concentrations up to 10 μM in HepG2 cells. We speculate that LDH assay is more sensitive to detect cytotoxicity than the resazurin assay and could indicate that HepG2 cells might be able to tolerate concentrations of Cr(VI) up to a toxic threshold. Thus, we suspect the presence of a protective mechanism to help HepG2 cells survive from Cr(VI) insult.

Mitochondrial biogenesis is one of the major compensating mechanisms in response to cellular stress. In the present study, mitochondrial biogenesis, comprising mtDNA copy number and mitochondrial mass, was increased, reaching a peak at 10 μM of Cr(VI). This event was also accompanied by upregulation of mRNA levels of *NDUFA1* and *NDUFB1*, *ATP5J* and *ATP5O*, and antioxidant enzymes, including *SOD1*, *SOD2* and *GSOT1*. At up to 20 μM of Cr(VI), mitochondrial biogenesis tended to decrease, exhibiting a concomitant increase in the mRNA expression of inflammatory cytokines and ROS, and inhibition of the antioxidant enzymes. These results indicate that mitochondrial biogenesis was adaptively upregulated to confront cellular stress at low concentrations of Cr(VI) in HepG2 cells. Small amounts of inflammatory cytokines and ROS production, which act as a retrograde nuclear signal, can improve mitochondrial biogenesis with adaptation to the stressor. Consistently, our results showed that Cr(VI) increased *TLR2/4* mRNA expression, and, subsequently, elevated ROS accumulation and *TNF* and *IL-1β* mRNA level in a concentration-dependent manner. Evidences have suggested that toll-like receptors (TLRs) play critical roles in the pathophysiological process of a variety of liver diseases, such as hepatitis B and hepatitis C [20]. TLR-2/4 activation leads to the production of pro-inflammatory cytokines IL-6 and TNF-α in hepatic NPCs and Hepatocytes [21]. Suliman et al. [22] demonstrated that, in the rat liver and heart, lipopolysaccharide stimulates mitochondrial biogenesis in response to inflammatory cell damage. They also revealed the simultaneous occurrence of mtDNA damage and compensatory mitochondrial biogenesis after exposure to *Escherichia coli* that resulted from the activation of TLR4 signaling pathways and nuclear factor-κB (NF-κB)-dependent cytokine production [22].

Slightly ROS accumulation and intense inflammation increase the transcriptional activity of NRF-1 and NRF-2 and induce expression of PGC-1α/β to promote detoxification through direct induction of antioxidant defenses, including superoxide dismutase 2 (SOD2), catalase and glutathione peroxidase [23,24]. In the present study, low concentrations of Cr(VI) increased *PGC-1α*, *SOD1* and *SOD2* mRNA level as well as *NRF-2* and *KEAP1* mRNA expression. Many genes encoding antioxidant enzymes are regulated by the NRF-2/KEAP1 pathway [10]. In addition, NRF-2 and its downstream factors can be activated by ROS to generate signals, resulting in co-induction of genes for mitochondrial biogenesis and countering inflammation. However, when the increasing toxicity of Cr(VI) overwhelms the cytoprotective capacity, the adverse effects could result in mitochondrial dysfunction and cell death [15]. It has been reported that mitochondrion is the sensitive target that can be perturbed by Cr(VI) [25]. Mitochondrial dysfunction is linked to oxidative stress and inflammatory response, and accumulating evidence has shown that mitochondrial dysfunction could be aggravated by increased mtROS generation, extracellular ATP and mtDNA release. This outcome was also demonstrated in the present study, as, at a high concentration of Cr(VI), mitochondrial biogenesis collapsed, accompanied by strong inhibition of *NDUFA1*, *NDUFB1*, *ATP5O* and *ATP5J* mRNA levels. Disturbance of mitochondrial function can induce cell apoptosis, and we evidenced that Cr(VI) caused apoptosis in a concentration-dependent increase. Cr(VI) induced oxidative stress and inflammatory response at low concentrations that triggered adaptively upregulated mitochondrial biogenesis and protective cell apoptosis. Nonetheless, when the adverse effects exceed the cell toxic tolerance, mitochondrial function collapses as a result of ATP depletion, and cell death switches to necrosis. This could be explained by the increased LDH release after Cr(VI) exposure, which is indicative of necrotic cell death [26].

PGC-1α/NRF-1/2-TFAM are the most acceptable regulators involved in mitochondrial biogenesis [27]. PGC-1α is the principal regulator of mitochondrial biogenesis that interacts with key nuclear transcription factors NRF-1 and NRF2, whose activation can trigger TFAM expression and then regulation of mtDNA replication and transcription [18]. In this study, both mRNA and protein levels of PGC-1α, NRF-1, NRF-2 and TFAM were upregulated at low concentration of Cr(VI) in HepG2 cells. In addition, we also observed that PGC-1α and NRF-1 translocated to the nucleus, and TFAM became highly expressed in the mitochondria. These data suggested that a certain extent of cellular stress can activate mitochondrial biogenesis by promoting PGC-1α, *NRF-1* and *TFAM* expression and translocation to protect the cell from Cr(VI) insult.

Mitochondrial biogenesis is regulated by multiple metabolic sensors, including silent information regulator-1 (SIRT1), MAPK, AKT, CREB, PTEN, among others [28]. We analyzed the potential involvement of sensors to regulate mitochondrial biogenesis, and found that low concentrations of Cr(VI) significantly elevated the mRNA levels of *SIRT1*, *CREB1*, *AKT1* and *FOXO1*, but not the levels of *AMPK1* and *PTEN*. A number of studies have reported that silent information SIRT1 deacetylation can activate PGC-1α [29] and is responsible for PTEN deacetylation. Li et al revealed that higher levels of SIRT1 was expressed in HCC specimens in comparison to normal liver tissues and cells, indicating that SIRT1 can facilitate hepatocellular carcinoma metastasis by upregulating mitochondrial biogenesis and PI3K/AKT signaling pathway [30]. PTEN can be regulated by ROS, which can also be inhibited by the enhanced ROS-dependent insulin signaling [31]. Consistent with our results, mRNA level of *PTEN* was decreased after exposure to Cr(VI), following the increased ROS production. PTEN can negatively regulate PI3K/AKT signaling pathway, a factor also demonstrated by the obtained data: When *PTEN* was decreased by Cr(VI), the mRNA level of *AKT1* was elevated at low concentrations of Cr(VI); but when the cells were exposed to higher concentrations, both mRNA levels of *PTEN* and *AKT1* were downregulated, probably as a consequence of the sub-cytotoxic effect exerted by this compound. The activation of AKT induces the phosphorylation of FOXO1, leading to the exclusion of this protein from the nucleus to the cytoplasm. Tobita et al reported that inhibition of SIRT1 could impair AKT/FOXO1 pathways, causing increased gluconeogenesis in human hepatocytes [32]. Puigserver et al. showed that PGC-1α interacted with and co-activated FOXO1 in hepatocytes [33]. Moreover, FOXO-PGC-1α interaction is known to be involved in oxidative stress protection in vascular endothelium [34,35]. Activation of the cAMP-CREB pathway results in phosphorylation of CREB (p-CREB), which binds the CREB-response element located proximal to the PGC-1α prompter, activating its transcription [36]. Consistent with the above evidence, SITR1/CREB1/ATK1 pathways might be activated to promote mitochondrial biogenesis against Cr(VI) insult. However, when mitochondrial biogenesis is collapsed by high concentration of Cr(VI), with the inhibition of protective mechanisms, the cells tended to die. In the present study, we only analyzed the mRNA levels of the factors potentially involved in mitochondrial biogenesis, but it would be interesting to investigate the deacetylation and phosphorylation levels of these factors in future studies to better understand the molecular mechanism involved in Cr(VI) cytotoxicity.

Therefore, we propose that mitochondrial biogenesis is a key adaptive mechanism to resist Cr(VI) hepatotoxicity. Addition of antioxidant supplements to the diet has been shown to maintain mitochondrial biogenesis to protect cells from death [37,38]. Previous studies reported that α-tocopherol exerted a protective role in Cr(VI)-induced oxidative damage [39,40,41]. Our results corroborate this finding as, at lower concentrations of Cr(VI), α-tocopherol inhibited the adaptive upregulated genes, maintaining the mitochondrial biogenesis within the normal range. This indicates that pretreatment with α-tocopherol could block the adaptive increasing mitochondrial biogenesis [42]. Truksa et al. reported that mitochondrially targeted α-tocopherol succinate could reduce the level of mtDNA transcripts in cancer cells but not in normal tissue [43], suggesting that α-tocopherol may depress the overexpression of mitochondrial biogenesis, which, in turn, could regulate mitochondrial homeostasis. However, α-tocopherol restored mitochondrial biogenesis at high concentrations of Cr(VI), indicating that α-tocopherol could protect cells from protect cells from Cr(VI)-induced damage through modulating mitochondrial biogenesis. Pretreatment with α-tocopherol resulted in a very strong protective role against Cr(VI)-induced hepatotoxicity and it seems that the possible mechanism involved might be similar to the mechanisms of other antioxidants, such as *N*-acetylcysteine (NAC) [44]. On the one hand, α-tocopherol effects could be attributed to extracellular reduction to prevent the cellular uptake of Cr(VI). Pretreatment of cells with α-tocopherol might also be able to participate in the intracellular reduction of Cr(VI), which is expected to elevate the level of intercellular α-tocopherol to increase the capacity of ROS scavenging and maintain mitochondrial homeostasis. Nevertheless, the mechanistic explanation of cellular responses to α-tocopherol is still unclear, and needs to be fully investigated.

## 4. Materials and Methods

### 4.1. Materials

Resazurin sodium salt, potassium dichromate (K_2_Cr_2_O_7_), α-tocopherol, and dimethyl sulfoxide (DMSO) were obtained from sigma (St. Louis, MO, USA). DMEM medium, and fetal bovine serum (FBS) were purchased from Gibco (Thermo Fisher, Waltham, MA, USA). All chemicals and solvents were analytical grade.

### 4.2. Cell Culture

Human hepatocellular carcinoma cells (HepG2) were kindly provided by Wang from the Cancer Research Center at Johns Hopkins University. HepG2 cells were maintained in DMEM medium containing 10% fetal bovine serum (FBS) and a 1% mixture of penicillin and streptomycin in a 5% CO_2_ humidified atmosphere at 37 °C. The medium was changed every other day, and the cells were passaged using trypsin.

### 4.3. Treatment and Cytotoxicity Assay

Cells (1 × 10^5^) were plated in the 24-well plate with 500 μL of medium, and were allowed to grow to 80%–90% confluence. HepG2 cells were exposed to different concentrations of K_2_Cr_2_O_7_ for 24 h. Cr(VI) working solution was prepared in Dulbecco’s Modified Eafle’s medium (DMEM) to reach final concentrations of 0, 5, 10, 20, 40, or 80 μM. Resazurin reduction assay was performed to determine cell viability after Cr(VI) treatment. Fifty microliters of resazurin (1 mg/mL stock) was added to the 24-well plates (500 μL/well), which were incubated for 2 h at 37 °C. Fifty microliters of color changed medium was transferred from each well to a 96-well plate and fluorescence was measured at 530 nm/590 nm (excitation/emission), using a multi-well fluorometric reader CytoFluor series 4000 (Applied Biosystems, Drive Foster, CA, USA). Lactate dehydrogenase (LDH) assay (Promega, Madison, WI, USA) was performed to determine cytotoxicity after Cr(VI) treatment. Fifty microliters of medium was transferred from each well to a 96-well plate and 50 μL of substrate mix was added to each well. The 96-well plate was then incubated for 30 min at room temperature. Subsequently, 50 μL of stop solution was added to each well and absorbance was measured at 560 nm using a plate reader. Data are presented as mean ± SEM. Three independent experiments in three replicates were performed.

### 4.4. Reactive Oxygen Species Measurement

Ten thousand cells were seeded into the 96-well plate with 100 μL of medium, and after reaching 80% confluence, they were treated with different concentrations of Cr(VI) for 24 h. Cells were washed three times with serum-free medium. One hundred microliters of medium containing CellROX**^®^** Green Reagent (Thermo Fisher Scientific, Waltham, MA, USA), at final concentration of 5 μM, was added to each well, and incubated for 30 min at 37 °C. The cells were washed twice with PBS and 100 μL of PBS was added to each well. The fluorescence was measured at 480/530 nm (excitation/emission) using a fluorometric reader (Applied Biosystems).

### 4.5. RNA Extraction and Quantitative Real-Time qPCR

One million cells were plated in the 6-well plate with 2 mL of medium, and after reaching 80% confluence, they were treated with different concentrations of Cr(VI) for 24 h. Total RNA was extracted after treatment using Trizol (Thermo Fisher Scientific). RNA quantity and purity were determined using NanoDrop 2000c (Thermo Fisher Scientific). Five hundred nanograms of RNA was reverse-transcribed using the M-MLV Promega Reverse Transcriptase (Promega, Madison, WI, USA), according to the manufacturer’s instructions. The expression of genes was evaluated using TaqMan gene expression assays or SYBR Green assays. Real time qPCR was performed using a 7500 Fast Real Time system machine (Applied Biosystems). Fold changes were calculated using the 2(-*C*t) method. GAPDH or 18 S was used as housekeeping gene for mRNA. The primer sequences were showed in Table 1 and Table 2.

### 4.6. Mito-Tracker Assay

One hundred thousand cells were plated in the 24-well plate with coverslip in 500 μL of medium, and after reaching 80% confluence, they were treated with different concentrations of Cr(VI) for 24 h. Mito-tracker probe (Thermo Fisher Scientific) was added to each well at 50 nM final concentration, then incubated for 45 min at 37 °C. Subsequently, the medium was removed and 500 μL of fresh medium was added to each well, and incubated for another 45 min at 37 °C. The cells were washed twice with warm PBS and fixed in 4% paraformaldehyde; then washed twice with PBS and blocked with blocking solution (10% goat serum, 1% BSA, 0.15% saponin in PBS), and co-incubated with TFAM primary antibody.

### 4.7. Measurement of Mitochondrial Mass

Ten thousand cells were seeded into the 96-well plate with 100 μL of medium, and after reaching 80% confluence, they were treated with different concentrations of Cr(VI) for 24 h. The mitochondrial mass was examined using the fluorescent probe 10-*N*-nonyl acridine orange (NAO). The cells were protected from light and incubated in medium containing 5 μM NAO for 30 min at 37 °C. NAO fluorescence intensity at 485 /530 nm (excitation/emission) was determined using a microplate Reader (Gemini EM, Molecular Devices, Sunnyvale, CA, USA).

### 4.8. Immunofluorescence

One hundred thousand cells were plated in the 24-well plate with coverslip in 500 μL of medium, and after reaching 80% confluence, they were treated with different concentrations of Cr(VI) for 24 h. Cells were fixed in 4% paraformaldehyde, washed twice in PBS and then blocked for 30 min in blocking solution (10% goat serum, 1% BSA, 0.15% saponin in PBS) at room temperature. They were incubated with primary antibody PGC-1α (1:50, Santa Cruz Biotechnology, Santa Cruz, CA, USA), NRF-1 (1:50, Santa Cruz Biotechnology), COXII (1:50, Santa Cruz Biotechnology) and TFAM (1:50, Santa Cruz Biotechnology) for 24 h at 4 °C; washed 3 times with PBS, and incubated with Goat anti-Mouse IgG/IgM(H+L) secondary antibody, Alexa Fluor 488, 1:500, or Goat anti-Rabbit IgG/IgM(H+L) secondary antibody, Alexa Fluor 568, 1:500, (Thermo Fisher Scientific) for another 1 h at room temperature. Nuclei were stained with Hoechst for 5 min, and mounted on slides with coverslips. Images were taken using a Zeiss UV-LSM 510 confocal microscope (Zeiss, Oberkochen, Germany).

### 4.9. Caspase-3/7 Staining

One hundred thousand cells were plated in the 24-well plate with coverslip in 500 μL of medium, and after reaching 80% confluence, they were treated with different concentrations of Cr(VI) for 24 h. Cells were washed twice with serum-free medium. Five hundred microliters of medium containing Caspase-3/7 Green Detection Regent (Thermo Fisher Scientific), at final concentration of 5 μM, was added to each well, and incubated for 30 min at 37 °C. The cells were washed twice with PBS, and 250 μL of 4% paraformaldehyde was added to the wells. The cells were then incubated for 20 min at room temperature. Nuclei were stained with Hoechst for 5 min, and mounted on slides with coverslips. Images were taken using a Zeiss UV-LSM 510 confocal microscope.

### 4.10. Western Blot Analysis

One million cells were plated in the 6-well plate with 2 mL of medium, and after reaching 80% confluence, they were treated with different concentrations of Cr(VI) for 24 h. The cells were collected for protein extraction using lysis buffer. An equal amount of concentrated proteins was loaded on 4–15% gradient SDS-polyacrylamide gel and transferred to a polyvinylidene difluoride membrane by electroblotting at 200 mA, 4 °C for 2 h. The membrane was blocked with 5% non-fat dry milk solution (TBS, 0.5% Tween-20, pH 7.4) for 1 h at 4 °C. The membrane was incubated with primary rabbit anti-PGC-1α monoclonal antibody (Santa Cruz Biotechnology) diluted 1:500, or goat anti-rabbit TFAM (1:500, Santa Cruz Biotechnology), NRF-1 antibody (1:500, Santa Cruz Biotechnology), and anti-GAPDH primary antibody (1:2000, Cell signaling Technology, Danvers, MA, USA) in blocking solution overnight at 4 °C. The membrane was washed thoroughly with TBST (50 mM Tris, 150mM NaCl, 0.1% Tween 20, PH 7.4) buffer and then incubated for 1 h with horseradish peroxidase-conjugated anti-rabbit IgG antibody (1:3000; Santa Cruz Biotechnology) in blocking solution, detected by chemiluminescence reagent plus, and exposed to film.

### 4.11. Statistical Analysis

Data were expressed as mean ± standard error of the mean (SEM). Comparisons of results were calculated by SPSS 19.0 software (version 19.0, IBM, Costa Mesa, CA, USA) using one-way analysis of variance, followed by the Fisher’s least significant difference (LSD) to compare the difference between subgroups. A level of *p* < 0.05 was considered significantly different.

## 5. Conclusions

Overall, the antioxidase system genes and mitochondrial biogenesis were upregulated at low concentrations of Cr(VI), indicating that a compensatory mechanism might be induced to defense the Cr(VI) insult. However, if the damage overwhelms the cell protective capacity, the antioxidase system genes and mitochondrial biogenesis could be collapsed by Cr(VI). However, pretreatment of α-tocopherol abolished excessive oxidative stress and inflammatory response, and maintained the mitochondrial biogenesis in balance, suggesting that maintenance of a homeostatic mitochondrial biogenesis level may be essential for survival of liver cells from Cr(VI) insult.

## Figures and Tables

**Table 1:** Primers sequences used in SYBER Green Expression Assays.

Gene Name	Forward Primer (5′-3′)	Reverse Primer (5′-3′)
*PGC-1a*	TCTGTGTCACTGTGGATTGGAG	AGTTCAGGAAGATCTGGGCAA
*Tfam*	TGCGGTTTCCCTTCATCTCC	ACTAGCGAGGCACTATGGGA
*NRF-1*	CCTGGTCCAGAACTTTACACAGA	CACTCCGTGTTCCTCCATGA
*NRF-2*	AACCAGTGGATCTGCCAACT	AAGTGACTGAAACGTAGCCG
*SIRT1*	GCGGTTCCTACTGCGCGA	TCACTAGAGCTTGCATGTGAGG
*MAPK1*	GCCGAAGCACCATTCAAGTT	GATGTCTGAGCACGTCCAGT
*mtDNA*	CAAACCTACGCCAAAATCCA	GAAATGAATGAGCCTACAGA
*AKT1*	CAGGATGTGGACCAACGTGA	AAGGTACGTTCGATGACAGT
*CREB1*	TTCAAGCCCAGCCACAGATT	AGTTGAAATCTGAACTGTTTGGAC
*PTEN*	CTCCCAGACATGACAGCCA	GCTTTGAATCCAAAAACCTTACTCA
*Caspase3*	TGGTTTTCGGTGGGTGTG	CCACTGAGTTTTCAGTGTTCTC
*Caspaase8*	AGTCTGTACCTTTCTGGCGG	TCTCCCAGGATGACCCTCTT
*GAPDH*	TGACAACAGCCTCAAGAT	GAGTCCTTCCACGATACC

**Table 2:** Primers sequences used in TaqMan^®^ Gene Expression Assays.

Assay Name	Assay ID	Catalog Number
*18S*	Hs99999901	4331182
*ATP5J*	Hs01081394_g1	4331182
*ATP5O*	Hs00919163	4331182
*NDUFA1*	Hs00915157_g1	4331182
*NDUFB1*	Hs00929425	4331182
*TLR2*	Hs02621280_s1	4331182
*TLR4*	Hs00152939_m1	4331182
*TNF*	Hs01113624_g1	4331182
*IL-1β*	Hs01555410_m1	4331182
*SOD1*	Hs00533490_m1	4331182
*SOD2*	Hs00167309_m1	4331182
*GSTO1*	Hs02383465_s1	4331182
*FOXO1*	Hs00231106_m1	4331182
*KEAP1*	hs01003430	4331182

## Abbreviations

AKT1	Threonine kinase 1
APT5O	ATP synthase, H^+^ transporting, mitochondrial F1 complex, O subunit
ATF2	Transcription factor
ATP5J	ATP synthase, H^+^ transporting, mitochondrial Fo complex subunit F6
Cr(VI)	Hexavalent chromium
CREB	cAMP response element-binding protein
FOXO1	Forkhead box class-O
GSOT1	Glutathione S-transferase omega 1
HepG2	Human Hepatocellular Carcinoma cells
IL-1β	Interleukin 1-β
KEAP1	Kelch like ECH associate protein 1
LDH	Lactate Dehydrogenase
MAPK1	Mitogen-activated protein kinase 1
mtDNA	Mitochondrial DNA
NDUFA1	NADH: ubiquinone oxidoreductase subunit A1
NDUFB1	NADH: ubiquinone oxidoreductase subunit B1
NRF	Nuclear respiratory factor
PGC-1α	Peroxisome-proliferator-activated receptor γ coactivator-1α
PTEN	Phosphatase and tensin homolog
ROS	Reactive oxygen species
SIRT1	Silent information regulator-1
SOD	Superoxide dismutase
TFAM	Mitochondrial transcription factor A
TLRs	Toll-like receptors
TNF	Tumor necrosis factor
